# A High-Speed and High-Saturation Output-Modified Uni-Traveling-Carrier Photodiode (MUTC-PD) with an Electric-Field Regulation Layer

**DOI:** 10.3390/s26123712

**Published:** 2026-06-11

**Authors:** Mengyu Zhang, Yuansen Shen, Kai Liu, Xiaofeng Duan, Yongqing Huang

**Affiliations:** State Key Laboratory of Information Photonics and Optical Communications, School of Electrical and Electronic Engineering, Beijing University of Posts and Telecommunications, Beijing 100876, China; zhangmengyu1@bupt.edu.cn (M.Z.); yuansen.shen@hotmail.com (Y.S.); xfduan@bupt.edu.cn (X.D.); yqhuang@bupt.edu.cn (Y.H.)

**Keywords:** electric-field regulation layer, high-speed response, RF output capability, peak drift velocity

## Abstract

To alleviate the trade-off between high-speed responses and RF output capability in modified uni-traveling-carrier photodiodes (MUTC-PDs), an MUTC-PD incorporating an electric-field regulation layer (EFRL-MUTC-PD) is proposed. A 20 nm EFRL is inserted between the PD’s collector layer and its cliff layer to tailor the electric-field distribution in the collector layer, thereby enabling electron transport near the peak drift velocity under high-photocurrent operation. Simulation results indicate that the optimal doping concentration of the EFRL is $1\times 10^{16}$ cm^−3^. For an 8 µm diameter device operated at a bias voltage of −4 V and a photocurrent of 15 mA, the simulation predicts a 3 dB bandwidth of 130 GHz and a transit-time-limited bandwidth of 162 GHz, corresponding to a 9.3% improvement in the simulated 3 dB bandwidth compared with a conventional MUTC-PD. In addition, the simulated RF output power reaches 11.54 dBm at 130 GHz under the adopted simulation assumptions.

## 1. Introduction

With the rapid development of fifth-generation (5G) communication systems and the expansion of sixth-generation (6G) technologies toward the millimeter-wave and terahertz bands, front-end devices are required to support broader bandwidth, lower latency, and higher transmission capacity [1,2]. Radio-over-fiber (RoF) technology combines the low loss and large bandwidth of optical fiber with the flexibility of wireless links and is therefore regarded as a promising solution for future high-frequency broadband access [3]. In RoF systems, photodiodes (PDs) are key devices for optical-to-electrical conversion. Their bandwidth determines the achievable carrier frequency, whereas their RF output capability strongly affects the transmission power and link distance [4]. Therefore, PDs simultaneously offering high bandwidth and high RF output capability are of considerable interest.

Uni-traveling-carrier photodiodes (UTC-PDs) and modified uni-traveling-carrier photodiodes (MUTC-PDs) rely primarily on electron transport. This feature relaxes the limitation imposed by hole transport on the frequency response and partially suppresses the space-charge effect, making these devices attractive for high-speed, high-power photodetection [5,6,7,8]. Previous studies have mainly improved MUTC-PD performance by optimizing the collector layer, the cliff layer, and device size. Xiong et al. proposed a double-drift-layer MUTC-PD and achieved a 3 dB bandwidth of 106 GHz with a responsivity of 0.165 A/W in a 6 µm device [9]. Dong et al. showed that optimization of the cliff-layer thickness improved the electric-field distribution under high optical power, leading to a simulated 3 dB bandwidth of 137 GHz and a saturated RF output power of 27.4 dBm at 60 GHz under −5 V bias [10,11]. Umezawa et al. reported a bandwidth exceeding 200 GHz in a 4 µm device by thinning the InGaAs/InP layer and reducing the junction capacitance [12].

Although these approaches are effective in enhancing bandwidth, they often do so at the expense of RF output capability, owing to reduced current-handling capability or electric-field collapse under high optical injection. As a result, the simultaneous improvement of bandwidth and RF output capability remains challenging. Xu et al. proposed an SMUTC-PD incorporating an electric-field regulation layer. By introducing a thin p-type layer behind the cliff layer to further optimize the electric field in the collector layer, a 16 µm device achieved a 3 dB bandwidth of 68 GHz and a responsivity of 0.502 A/W under a low optical power of 500 W/cm^2^ (1 mW) [13]. However, that study mainly focused on low-optical-injection conditions. Its influence on the bandwidth and RF output capability under high optical power remains unclear.

To address this issue, this paper proposes an EFRL-MUTC-PD incorporating a 20 nm EFRL between the collector layer and the cliff layer. By optimizing the doping concentration of this layer under high optical injected power operation, the electric-field distribution and electron transport in the collector layer are improved. The PD’s working scheme is carefully analyzed and its performance is optimized by simulations.

## 2. Device Operating Principle Analysis and Performance Optimization

### 2.1. Operating Principle

Under low optical power, the influence of the photocurrent on the internal electric-field distribution of an MUTC-PD is relatively weak, and the field is mainly determined by the applied bias and epitaxial structure. Under high-photocurrent operation, however, the photo-generated carriers redistribute the electric field in the collector layer. This redistribution directly affects the electron drift velocity, carrier transit time, high-speed response and RF output capability of the PD.

In InP, the electron drift velocity does not increase monotonically with the electric-field intensity. Instead, it reaches a peak at approximately 12 kV/cm and then gradually decreases as the field intensity further increases. For MUTC-PDs, better performance can be achieved by maintaining the electric-field range near the value corresponding to the peak electron drift velocity. Compared with the local velocity-overshoot effect, this mechanism is more suitable for a long collector layer and is therefore more relevant to balancing high-speed responses and RF output capability.

Under high-photocurrent conditions, the electric-field distribution should be analyzed using the Poisson equation, including the photocurrent-density term:

(1) $$\frac{dE}{dx}=\frac{q}{\varepsilon }\left[N\left(x\right)-\frac{J_{c}}{qv_{n}}\right]$$

where *E* is the electric-field intensity, *x* is the position along the epitaxial growth direction, *q* is the elementary charge, ε is the dielectric permittivity of the semiconductor material, and N(x) is the position-dependent net ionized doping concentration. $J_{c}$ denotes the photocurrent density, and $v_{n}$ is the electron drift velocity. This relation indicates that, as the photocurrent density increases, the electric field in the collector layer is redistributed and may deviate from the designed distribution. Carrier accumulation screens the local electric field and triggers the Kirk effect [14]. As a result, a low-field region may appear in the collector layer, reducing the electron drift velocity, increasing the carrier transit time, and degrading both the frequency response and RF output power. Therefore, under high-current operation, optimizing only the conventional structural parameters is often insufficient to maintain a stable and favorable electric-field distribution in the collector layer.

The saturation current density of the MUTC-PD represents the maximum effective photo-generated current density that can be sustained under high-photocurrent conditions and can be expressed as follows:

(2) $$J_{Cmax}=qV_{sclimit}N_{DC}$$

where $V_{sclimit}$ is the saturation electron velocity, while $N_{DC}$ is the doping concentration in the PD’s depletion region. In general, the saturation current density can be improved either by increasing the donor concentration in the collector layer or by increasing the effective electron transport velocity. However, increasing collector-layer doping may weaken the internal electric field and degrade the breakdown characteristics. In contrast, optimizing the electric-field distribution so that electrons travel close to the peak drift velocity over a longer distance provides a more effective way to improve the current-handling capability while preserving high-speed performance.

### 2.2. Epitaxial Structure Design

**Figure 1:**
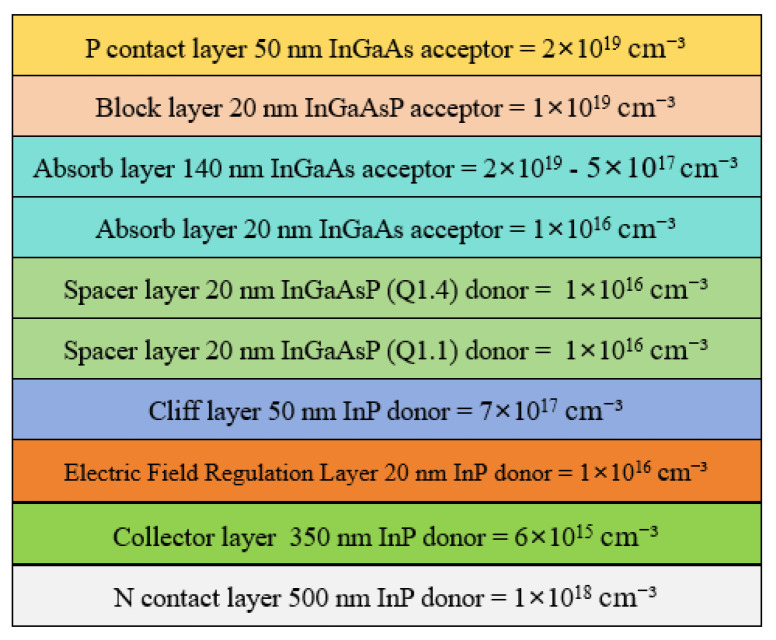
Epitaxial structure of the proposed EFRL-MUTC-PD.

Based on the above analysis, an EFRL-MUTC-PD structure is proposed, as shown in Figure 1. The device diameter is set to 8 µm in the simulation. The top-down epitaxial layers’ parameters of the EFRL-MUTC-PD are as follows: p-contact layer (50 nm In_0.53_Ga_0.47_As, $2\times 10^{19}$ cm^−3^), blocking layer (20 nm In_0.649_Ga_0.351_As_0.755_P_0.245_, $1\times 10^{19}$ cm^−3^), p-type absorption layer 1 (140 nm In_0.53_Ga_0.47_As, from $1\times 10^{19}$ cm^−3^ to $5\times 10^{17}$ cm^−3^), absorption layer 2 (20 nm In_0.53_Ga_0.47_As, $1\times 10^{16}$ cm^−3^), spacer layer 1 (20 nm In_0.680_Ga_0.320_As_0.691_P_0.309_, Q1.4, $1\times 10^{16}$ cm^−3^), spacer layer 2 (20 nm In_0.863_Ga_0.137_As_0.300_P_0.700_, Q1.1, $1\times 10^{16}$ cm^−3^), cliff layer (50 nm InP, $7\times 10^{17}$ cm^−3^), electric-field regulation layer (20 nm InP, $1\times 10^{16}$ cm^−3^), collector layer (350 nm InP, $6\times 10^{15}$ cm^−3^), and n-contact layer (500 nm InP, $1\times 10^{18}$ cm^−3^).

Compared with a conventional MUTC-PD, the proposed device introduces a 20 nm EFRL between the collector layer and the cliff layer. This additional layer provides an extra degree of freedom for tuning the electric-field distribution in the collector layer under high-photocurrent operation. As a result, the electric field intensity can be shifted toward the range favorable for near-peak electron drift velocity, which is beneficial for improving both bandwidth and RF output capability.

The results presented in this paper were obtained using Silvaco Atlas simulations. The Fermi–Dirac carrier statistical model, the Shockley–Read–Hall recombination model, the Auger recombination model, the analytic concentration-dependent mobility model, the parallel electric-field-dependent mobility model, and the negative differential mobility model were used in the simulations. The load and series resistances were set to 50 Ω and 15 Ω, respectively, and a 5 fF parasitic capacitance from the metal electrodes was included. The optical and electrical parameters of InP and InGaAs were taken from the literature [15,16], while the mobility-related fitting parameters are summarized in Table 1 according to the experimental data in [17].

## 3. Simulation Results and Discussion

To evaluate the proposed design, simulations were first performed on a conventional MUTC-PD to clarify the effects of the cliff-layer parameters on the electric-field distribution and device performance under high-photocurrent conditions. The EFRL was then introduced into the EFRL-MUTC-PD, and its doping concentration was optimized. The analysis focuses on the electric-field distribution, electron velocity, transit-time-limited bandwidth, 3 dB bandwidth, capacitance, and RF output power.

**Figure 2:**
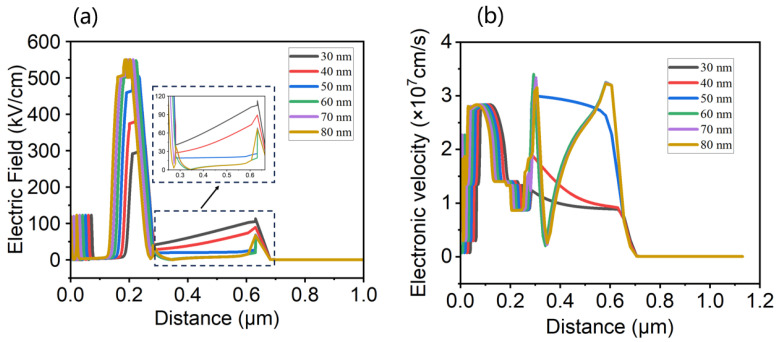
Simulated distributions of (**a**) the electric field and (**b**) electron velocity for the conventional MUTC-PD with different cliff-layer thicknesses.

To establish a reference for comparison, the cliff-layer thickness of the conventional MUTC-PD was optimized under high-photocurrent conditions, with the optimized cliff-layer doping concentration of $7\times 10^{17}$ cm^−3^ adopted in the simulation. Figure 2 shows the simulated distributions of the electric field and electron velocity for the conventional MUTC-PD with different cliff-layer thicknesses. Under a bias voltage of −4 V and a photocurrent of 15 mA, increasing the cliff-layer thickness strengthens the local electric field near the cliff layer but reduces the overall electric field in the collector layer. When the cliff-layer thickness is 50 nm, the electric field in the collector layer remains relatively uniform at approximately 18 kV/cm, resulting in a high and smooth electron-velocity distribution. A further increase in thickness leads to local low-field regions and a less uniform electron-velocity distribution, which are unfavorable for high-speed operation.

**Figure 3:**
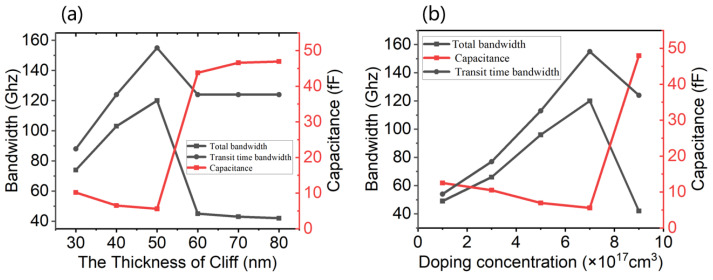
Dependence of the frequency response on (**a**) the cliff-layer thickness and (**b**) the cliff-layer doping concentration.

Figure 3 summarizes the effects of the cliff-layer thickness and doping concentration on the frequency response. In both cases, the 3 dB bandwidth first increases and then decreases, whereas the capacitance exhibits the opposite trend. These results indicate that both insufficient and excessive cliff-layer thicknesses or doping concentrations degrade the high-frequency performance. The conventional MUTC-PD achieves the best overall performance with a cliff-layer thickness of 50 nm and a doping concentration of $7\times 10^{17}$ cm^−3^, corresponding to a 3 dB bandwidth of 119 GHz under a bias voltage of −4 V and a photocurrent of 15 mA. Although optimization of the cliff layer improves the bandwidth, the response remains sensitive to parameter variation, indicating limited design tolerance.

To further improve the electric-field tolerance on the PD’s parameters and enhance the design flexibility, an EFRL was introduced between the collector layer and the cliff layer.

**Figure 4:**
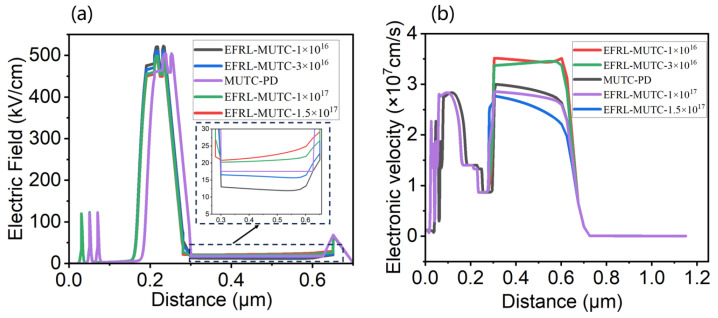
Simulated distributions of (**a**) the electric field and (**b**) electron velocity for the EFRL-MUTC-PD with different doping concentrations of the electric-field regulation layer.

Figure 4 shows the simulated distributions of the electric field and electron velocity for the EFRL-MUTC-PD with different doping concentrations of the EFRL. After the EFRL is introduced, the electric field in the collector layer varies with the doping concentration of this layer. In the conventional MUTC-PD, the electric field in the collector layer is approximately 18 kV/cm at a photocurrent of 15 mA. When the doping concentration of the EFRL is $1\times 10^{16}$ cm^−3^, the electric field in the collector layer decreases to approximately 12 kV/cm, which is closer to the field range corresponding to the peak electron drift velocity in InP. At the same time, the electron velocity becomes higher and more uniform across the collector layer. These results suggest that a properly designed EFRL can improve carrier transport by reshaping the electric-field distribution under high-photocurrent operation.

For the 8 µm diameter device, the responsivity is approximately 0.17 A/W, corresponding to a quantum efficiency of 13.7% at 1550 nm. Under the simulated optical intensity of $1.74\times 10^{5}$ W/cm^2^, the photocurrent is approximately 15 mA, corresponding to a photocurrent density of $2.98\times 10^{4}$ A/cm^2^. The collector-layer electric field is approximately 12 kV/cm, which is much lower than the critical electric field of InP (500 kV/cm), indicating a large breakdown margin under the simulated operating condition.

**Figure 5:**
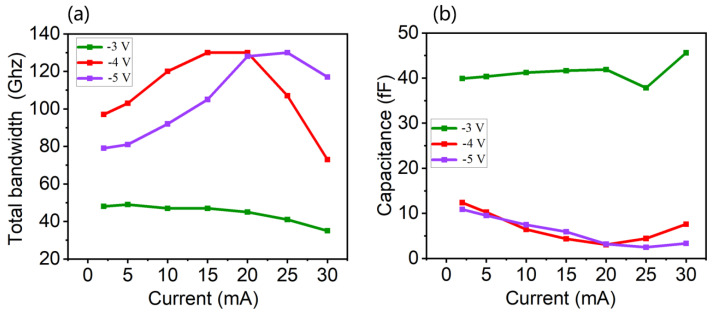
Dependence of (**a**) the total 3 dB bandwidth and (**b**) capacitance on the photocurrent under different reverse biases.

Figure 5 shows the dependence of the 3 dB bandwidth and capacitance on the photocurrent under different biases. With increasing photocurrent, the 3 dB bandwidth first increases and then decreases, whereas the capacitance initially decreases and then rises again at high photocurrent. In the simulation, the optimum performance is observed at −4 V, where the 3 dB bandwidth remains above 100 GHz over the photocurrent range from 5 mA to 25 mA and is predicted to reach a maximum of approximately 130 GHz. This behavior can be explained by the working mechanism discussed based on the electric-field analysis in Figure 4. Under high-photocurrent operation, the Kirk effect redistributes the electric field in the collector layer. At a bias voltage of −4 V, the collector layer can be fully depleted under large photocurrent, and the electric-field intensity in the collector layer falls within the range corresponding to the peak electron drift velocity in InP. Therefore, electron transport in the collector layer becomes most efficient, the transit-time-limited bandwidth reaches its maximum, and the device capacitance remains at a relatively low level, leading to the optimum overall bandwidth. In contrast, at −3 V, the electric field in the collector layer is insufficient under large photocurrent, resulting in reduced bandwidth and relatively large capacitance. When the bias is further increased to −5 V, the collector layer remains depleted; however, the electric-field intensity deviates from the optimal range for peak electron drift velocity. Therefore, the overall bandwidth is not further improved.

**Figure 6:**
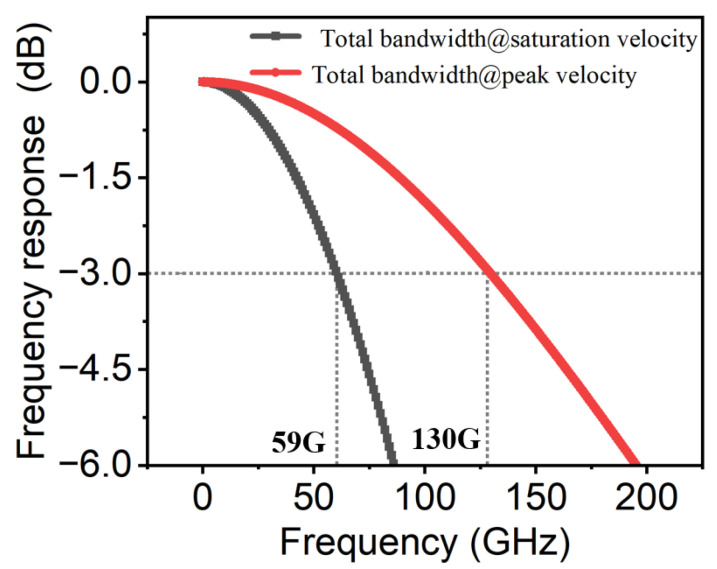
Frequency responses of the EFRL-MUTC-PD under different electron drift velocities in the collector layer. (The grey dotted line indicates the −3 dB frequency-response degradation level.)

To further clarify the role of the peak electron drift velocity, the frequency responses of the EFRL-MUTC-PD were compared under two electron-transport regimes in the collector layer, corresponding to the saturation-velocity and peak-drift-velocity regimes. These two electron-transport regimes were implemented by tuning the electric-field distribution in the collector layer through different bias voltages, rather than by changing the mobility model parameters. The same field-dependent mobility model and material parameters were used in both cases. Under a bias voltage of −4 V, the collector-layer electric field is approximately 12 kV/cm, corresponding to the near-peak-drift-velocity regime. In contrast, under a bias voltage of −8 V, the collector-layer electric field increases to approximately 80 kV/cm, and electron transport falls into the high-field saturation-velocity regime. As shown in Figure 6, the simulated frequency response was compared under two electron-transport conditions. When electrons drift at the saturation velocity, the 3 dB bandwidth is approximately 59 GHz. In contrast, when the electric field in the collector layer is adjusted to support near-peak electron drift velocity, the simulated 3 dB bandwidth increases to approximately 130 GHz. This improvement originates from the reduced carrier transit time, since the transit time is inversely proportional to the electron drift velocity. Therefore, regulating the collector-layer electric field to maintain electrons near the peak drift velocity is beneficial for improving the high-speed response of the EFRL-MUTC-PD.

**Figure 7:**
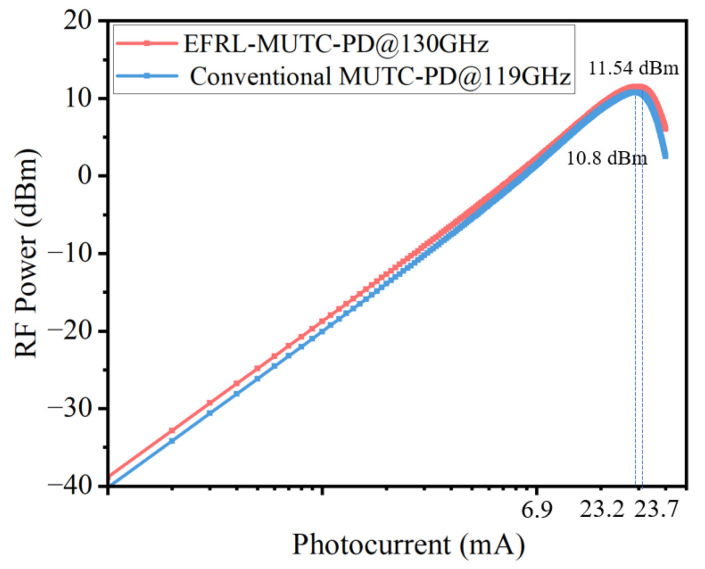
Simulated RF output power of the conventional MUTC-PD and the optimized EFRL-MUTC-PD under a bias voltage of −4 V.

Figure 7 compares the simulated RF output power characteristics of the optimized conventional MUTC-PD and the proposed EFRL-MUTC-PD under a bias voltage of −4 V. For both devices, the RF output power increases with photocurrent and gradually approaches saturation at high photocurrent. The conventional MUTC-PD is evaluated at 119 GHz because this frequency corresponds to its simulated maximum 3 dB bandwidth, whereas the optimized EFRL-MUTC-PD is evaluated at 130 GHz because this frequency corresponds to the simulated maximum 3 dB bandwidth predicted for the proposed structure. Therefore, the RF output powers are compared at the respective maximum-bandwidth frequencies of the two devices to evaluate their high-speed RF output capability near their bandwidth limits.

Under the adopted simulation assumptions, the simulated RF output power of the conventional MUTC-PD reaches approximately 10.80 dBm at 119 GHz. In comparison, the optimized EFRL-MUTC-PD is predicted to reach a simulated RF output power of 11.54 dBm at 130 GHz. This improvement can be attributed to the introduction of the EFRL. Under high-photocurrent operation, the EFRL adjusts the electric-field distribution in the collector layer toward the range favorable for near-peak electron drift velocity. As a result, the carrier transit time is shortened, carrier accumulation and space-charge screening are alleviated, and the effective depletion condition of the collector layer is improved. These effects help maintain the RF photocurrent response at high optical injection levels, thereby partially alleviating the trade-off between high-speed responses and RF output capability.

To provide a more comprehensive comparison with representative reported MUTC-PD results, Table 2 summarizes the device diameter, operating condition, responsivity, 3 dB bandwidth, RF output power, and result type. It should be noted that the listed devices were designed for different application targets and were evaluated under different diameters, bias voltages, photocurrents or optical input powers, and RF operating frequencies. Therefore, the RF output powers should not be interpreted as a strict one-to-one comparison. As shown in Table 2, smaller-diameter devices generally exhibit higher bandwidth but relatively limited RF output capability, whereas larger-diameter devices usually provide higher RF output power at the expense of bandwidth. Under the adopted simulation assumptions, the proposed EFRL-MUTC-PD achieves a simulated 3 dB bandwidth of 130 GHz and an RF output power of 11.54 dBm at 130 GHz, indicating a favorable balance between high-speed responses and RF output capability at a high operating frequency.

## 4. Conclusions

This paper proposes an EFRL-MUTC-PD incorporating a 20 nm EFRL between the collector layer and the cliff layer. By optimizing the doping concentration of the EFRL, the electric field in the collector layer can be shifted toward the range corresponding to the peak electron drift velocity in InP, thereby improving electron transport under high-photocurrent operation. Simulation results show that the optimal doping concentration of the EFRL is $1\times 10^{16}$ cm^−3^. Under a bias voltage of −4 V and a photocurrent of 15 mA, the 8 µm diameter device is predicted to achieve a 3 dB bandwidth of 130 GHz, a transit-time-limited bandwidth of 162 GHz, and a simulated RF output power of 11.54 dBm at 130 GHz. Compared with the conventional MUTC-PD, the proposed structure is expected to provide a better balance between high-speed responses and RF output capability, indicating its potential for the design of high-frequency, high-power photodetectors.

## Figures and Tables

**Table 1:** Material parameters used in the simulation.

Parameter	InP	InGaAs
Electron mobility, $\mu_{n}$	5400 cm^2^/Vs	12,000 cm^2^/Vs
Hole mobility, $\mu_{p}$	200 cm^2^/Vs	300 cm^2^/Vs
Conduction band density of states, $N_{c}$	$1.1\times 10^{19}$ cm^−3^	$7.7\times 10^{18}$ cm^−3^
Valence band density of states, $N_{v}$	$5.7\times 10^{17}$ cm^−3^	$2.1\times 10^{17}$ cm^−3^
Electron saturation velocity	$8.65\times 10^{6}$ cm/s	$8.35\times 10^{6}$ cm/s
Hole saturation velocity	$5\times 10^{6}$ cm/s	$5\times 10^{6}$ cm/s
Electron and hole lifetime	$2\times 10^{-9}$ s	$1\times 10^{-7}$ s
Electron Auger coefficient	$3.7\times 10^{-31}$ cm^6^/s	$3.2\times 10^{-28}$ cm^6^/s
Hole Auger coefficient	$8.7\times 10^{-30}$ cm^6^/s	$3.2\times 10^{-28}$ cm^6^/s
Real refractive index (1550 nm)	3.2	3.51
Imaginary refractive index (1550 nm)	0	0.106

**Table 2:** Comparison of representative MUTC-PD results.

Device	Diameter (µm)	Operating Condition	Responsivity (A/W)	3 dB Bandwidth (GHz)	RF Output Power	Result Type
Xu et al. [13]	16	−3 V; 1 mW	0.502	68	Not reported	Simulation
Rouvalis et al. [18]	20	−5 V; 74 mA	0.4	30	17 dBm@30 GHz	Experiment
Xiong et al. [19]	5	−3 V; 20 mW	0.15	172	Not reported	Simulation
Han et al. [20]	6	−3 V; 16 mA	0.51	102	2.7 dBm@100 GHz	Experiment
This work	8	−4 V; 15 mA	0.17	130	11.54 dBm@130 GHz	Simulation

## Data Availability

The original contributions presented in this study are included in the article.

## References

[1] Wang C., Wang J., Hu S., Jiang Z., Tao J., Yan F. (2021). Key Technologies in 6G Terahertz Wireless Communication Systems: A Survey. IEEE Veh. Technol. Mag..

[2] Navaratna N., Tan Y.J., Kumar A., Gupta M., Singh R. (2023). On-Chip Topological THz Biosensors. Appl. Phys. Lett..

[3] Soni M.K., Goel A., Gupta Y.K. (2024). A Comprehensive Overview of Advancing Radio over Fiber (RoF) Technology: Challenges, Solutions Strategies for Future Prospects for High-Performance Connectivity. Proceedings of the 2024 IEEE 2nd International Conference on Innovations in High Speed Communication and Signal Processing (IHCSP).

[4] Jia S., Lo M.-C., Zhang L., Ozolins O., Udalcovs A., Kong D., Pang X., Guzman R., Yu X., Xiao S. (2022). Integrated Dual-Laser Photonic Chip for High-Purity Carrier Generation Enabling Ultrafast Terahertz Wireless Communications. Nat. Commun..

[5] Ishibashi T., Fushimi H., Ito H., Furuta T. (1999). High Power Uni-Traveling-Carrier Photodiodes. Proceedings of the International Topical Meeting on Microwave Photonics. MWP’99. Technical Digest.

[6] Zhang J., Zhang J., Wang Q., Chen J., Hua B., Cai Y., Lei M., Zhu M. (2023). Experimental Comparison of Commercial PIN-PD and UTC-PD for THz Power and Transmission Performance in the 370 GHz–430 GHz Band. Proceedings of the 2023 Opto-Electronics and Communications Conference (OECC).

[7] Nellen S., Ishibashi T., Deninger A., Kohlhaas R.B., Liebermeister L., Schell M., Globisch B. (2020). Experimental Comparison of UTC- and PIN-Photodiodes for Continuous-Wave Terahertz Generation. J. Infrared Millim. Terahertz Waves.

[8] Tian Y., Xiong B., Sun C., Hao Z., Wang J., Wang L., Han Y., Li H., Gan L., Luo Y. (2023). Ultrafast MUTC Photodiodes over 200 GHz with High Saturation Power. Opt. Express.

[9] Chao E., Xiong B., Sun C., Hao Z., Wang J., Wang L., Han Y., Li H., Yu J., Luo Y. (2022). D-Band MUTC Photodiodes with Flat Frequency Response. IEEE J. Sel. Top. Quantum Electron..

[10] Dong X., Liu K., Huang Y., Duan X., Wang Q., Ren X. (2023). Design of High-Speed UTC-PD with Optimization of Its Electron Transit Performance and Parasitic Capacitance. IEEE Photonics J..

[11] Dong X., Liu K. (2024). Modified Uni-Traveling-Carrier Photodetector with Its Optimized Cliff Layer. Sensors.

[12] Umezawa T., Nakajima S., Matsumoto A., Akahane K., Yamamoto N. (2023). Ultra-Broadband UTC-PD Using Well-Optimized InGaAs/InP Active Layer Toward 200-GHz Bandwidth and Beyond. Proceedings of the 2023 Conference on Lasers and Electro-Optics Europe & European Quantum Electronics Conference (CLEO/Europe-EQEC).

[13] Xu J., Liu K., Dong X., Duan X., Huang Y., Wang Q., Ren X. (2025). Design of High-Speed MUTC-PD with Electric Field Regulation Layer. Chin. Opt..

[14] Kirk C.T. (1962). A Theory of Transistor Cutoff Frequency (fT) Falloff at High Current Densities. IRE Trans. Electron Devices.

[15] Adachi S. (1992). Physical Properties of III-V Semiconductor Compounds.

[16] Zang G., Huang Y., Ren X., Duan X., Cai S., Zhang X., Wang Q., Wang J. (2013). Numerical Simulation of the Uni-Traveling Carrier Photodiode with GaAsSb/InP Heterojunction. Proceedings of the Asia Communications and Photonics Conference 2013.

[17] Srivastava S. (2003). Simulation Study of InP-Based Uni-Traveling Carrier Photodiode. Ph.D. Thesis.

[18] Rouvalis E., Baynes F.N., Xie X., Li K., Zhou Q., Quinlan F., Fortier T.M., Diddams S.A., Steffan A.G., Beling A. (2014). High-Power and High-Linearity Photodetector Modules for Microwave Photonic Applications. J. Light. Technol..

[19] Xiong W., Peng Z., Yao R., Guo Q., Chi C., Ji C. (2023). Systematic Analysis of a Modified Uni-Traveling-Carrier Photodiode under High-Power Operating Conditions. Photonics.

[20] Han Y., Tian Y., Xiong B., Sun C., Wang J., Hao Z., Han Y., Wang L., Li H., Gan L. (2024). Double-Cliff-Layer Uni-Traveling-Carrier Photodiode with High Responsivity and Ultra-Broad Bandwidth. Chin. Opt. Lett..

